# Self-regulated spacing in a massive open online course is related to better learning

**DOI:** 10.1038/s41539-020-0061-1

**Published:** 2020-03-16

**Authors:** Paulo F. Carvalho, Faria Sana, Veronica X. Yan

**Affiliations:** 1 Human-Computer Interaction Institute, Carnegie Mellon University, 5000 Forbes Ave, Pittsburgh, PA 15213 USA; 2 Centre for Psychology, Athabasca University, 1 University Dr. Athabasca, Alberta, T9S 3A3 Canada; 3 Department of Educational Psychology, The University of Texas at Austin, 1912 Speedway, Ste 504, Austin, TX 78712 USA

**Keywords:** Education, Human behaviour, Education, Human behaviour

## Abstract

In this study, we examined students’ natural studying behaviors in massive, open, online course (MOOC) on introductory psychology. We found that, overall, distributing study across multiple sessions—increasing spacing—was related to increased performance on end-of-unit quizzes, even when comparing the same student across different time-points in the course. Moreover, we found important variation on who is more likely to engage in spaced study and benefit from it. Students with higher ability and students who were more likely to complete course activities were more likely to space their study. Spacing benefits, however, were largest for the lower-ability students and for those students who were less likely to complete activities. These results suggest that spaced study might work as a buffer, improving performance for low ability students and those who do not engage in active practices. This study highlights the positive impact of spacing in real-world learning situations, but more importantly, the role of self-regulated learning decisions in shaping the impact of spaced practice.

## Introduction

Being able to recall and apply previously learned information is key for successful learning. In laboratory settings, several strategies show promise to improve learning. One such strategy is to distribute or space out learning. The spacing effect is the finding whereby long-term memory is enhanced when study time is distributed across multiple learning sessions instead of massed into a single learning episode^1–3^. In other words, if a student were to devote 10 h of study to a particular topic, it is better to spread those hours out across multiple shorter learning sessions than to try to do all their studying in only one or two longer learning sessions. Since the spacing effect was first demonstrated more than a century ago^2^, hundreds of studies have replicated and extended its benefits to learning across numerous domains and age groups, both in laboratory settings, and recently, in small-scale classroom studies (for reviews see ref. ^4–9^).

In a typical spacing study paradigm, participants are exposed to two or more study sessions, with an interval of time (lag) separating the different study sessions of the same materials, and some retention interval (test delay) separating the last study session and a final test. Massed study is when there is no lag such that the study sessions occur back-to-back, and spaced study is when the study sessions are separated by some amount of time (ranging anywhere from a few seconds to several weeks). Typical findings show that spaced study, leads to better recall on a final test, particularly when the test is delayed (for reviews see ref. ^10^).

There are, however, only a limited number of studies that have examined whether such strategies scale up to real world educational situations particularly when students are in control of their learning. Why is this important? Laboratory studies tend to use simplified stimuli such as word lists, and are under conditions that are tightly controlled by the experimenter^10^. In most studies, spacing is typically defined as some form of temporal lag in-between repeated occurrences of the same study content. However, students typically do not study identical content, but rather content that is related to the same topic. Moreover, while learning in classroom settings involves large timescales, complex stimuli, and is often under the control of the learner, laboratory studies often involve compressed timescales in which the learners have limited time to learn the material and do not have to maintain it for long periods of time. To make practical use of the spacing effect in the classroom, it must be shown that the spacing effect operates at educationally meaningful timescales. Several studies support a non-monotonic effect of lags in which recall improves with increasing lags until they become “too long”, followed by a reduction in recall with a further increasing lag^11–13^. In other words, when we consider whether students are spacing optimally, it may also be important to consider the retention interval. The optimal amount of spacing depends on the retention interval (RI)—the longer the retention interval, the longer the optimal spacing. For example, Cepeda et al.^13^ systematically varied the spacing interval and the retention interval for learning obscure facts, and found that the optimal spacing gap for a one-week test delay is about one day, while the optimal spacing gap for a one-year test delay is about three weeks.

To what extent is spaced study in complex educational settings related to student performance? Most evidence of the spacing effect comes from situations where learners are not in control of their learning. For example, Lindsey, Shroyer, Pashler, and Mozer^14^ showed that when students in a language class spaced their practice according to a schedule, their long-term performance in the course was better than if they massed their practice. However, self-direction can change how learning works^15^ and might have an effect on how spacing affects learning and its impact on learning outcomes^16^. For example, in a lab study, Ciccone and Brelsford^16^ demonstrated that the relative benefits of spacing differed between a situation when the learner was in control of spacing/study pace and a situation where they were not (see also ref. ^17^ for similar evidence in a yoked-design classroom study). Similarly, Tullis and collaborators^18,19^ have demonstrated that not honoring learners self-regulated study choices resulted in worse learning. Importantly, there are several potential mechanisms for why this would be the case. Gureckis and Markant^15^ suggest that self-regulated learning changes the task substantially because learners can take data-driven approaches to optimize information search, which might apply different amounts of effort than directed instruction, and might have different inductive and sampling assumptions. Although our work cannot disambiguate among these possibilities—and was not designed to— this previous research does make it clear that self-regulation can have an impact on strategy use and learning that is currently not well-understood. The gap between how we learn and what learners do is an important one to close, because most studying is self-regulated—this is especially true for post-secondary learning, and increasingly true in K-12 education^20–22^. This need for effective self-regulation is particularly important in online classes.

How do people choose to incorporate spacing in their own study practices, how do these choices vary across individuals, and how are these choices related to learning outcomes? We know that spacing has benefits for long-term memory and memory maintenance from large scale studies of language learning^23^, but we do not know how these benefits scale up to more naturalistic settings. For example, we do not currently understand if and to what extent spaced study improves learning of complex materials where overlap from one event to another might be only partial or not perceptually salient. Moreover, we do not know how these choices might vary. Theories of spacing suggest that optimal spacing intervals should depend on learners’ prior knowledge or ability^24^ and on retention interval between study and test^13^. Although we cannot directly test these theories, we can examine how individuals’ self-directed spacing choices vary by prior knowledge or ability and subsequent retention interval between final study and quizzes.

Previous research using data from real-world situations has also focused on how completing activities during study promotes learning^25^, and the benefits of active engagement in online courses^26^, but often ignores the potential for improving learning even in relatively passive situations. It is possible that practice can be complemented by spacing, such that, even when students cannot or decide not to practice during study, spacing offers an alternative strategy through which they can still engage in active learning. Addressing these gaps in the literature will allow us to establish the external validity of the phenomena and promote its applicability to real-world situations.

Despite abundant evidence in the cognitive psychology literature in favor of spaced learning, students do not always appreciate the benefits of spacing^27^. For example, in the lab, by simply changing the conditions of a task, researchers can make students appear to make the right decision to space their study^28–31^ or to make the wrong decision to mass their study^32,33^. When asked directly about what they do or would do in their own learning, surveys show that people report that they tend not to return to previously studied materials^34–38^.

A less studied aspect, however, is the extent to which self-regulated study decisions are shaped by different students characteristics. Do students with different prior knowledge or ability levels, for example, make different self-regulated spacing decisions? One possible hypothesis is that higher-achieving students make more effective study choices. For example, Hartwig and Dunlosky^34^ found that students with higher grade point averages (GPA) were less likely than those with lower GPA to report that their study decisions were driven by deadlines, and were less likely to study late at night. They did not, however, find that self-reported spacing behavior was related to GPA. Students with higher working memory use efficient strategies (e.g., controlled attention to task-relevant goals, cue-driven retrieval, and integration from long-term memory) to process information^39^. In fact, lower working memory capacity individuals benefit more from instructional treatments than their counterparts with retrieval practice^39–41^, and multimedia instruction e.g., ^42,43^ because it forced them to use strategies that they normally would not.

In the present study, we examine how self-regulated spacing is used by students in an online environment and how it relates to their learning outcomes, using data from a Massive Online Open Course (MOOC). A MOOC presents the perfect opportunity to study self-regulated spacing: students care about what they are learning, the material is novel and relatively difficult, and the online learning system can track actual student behaviors instead of relying on self-reported use of spacing.

## Results

**Fig. 1:**
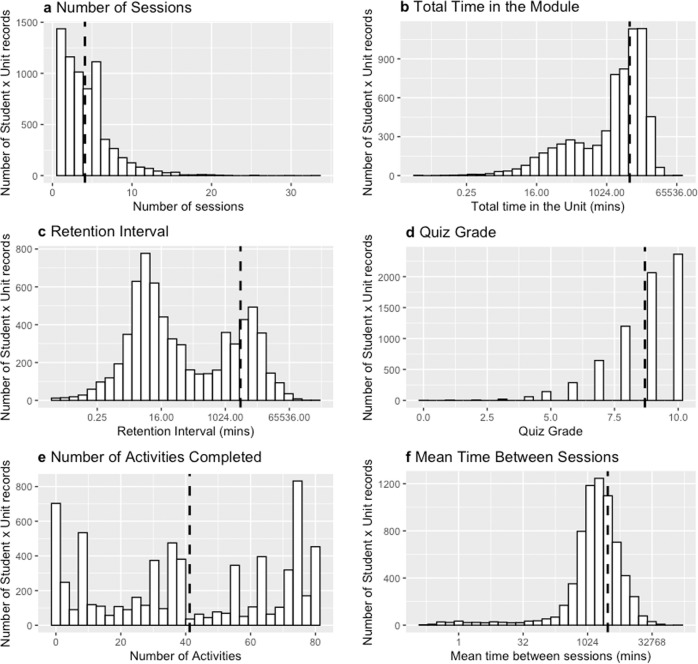
Description of study behaviors. Histograms detailing the distribution of **a** number of sessions spent on each unit, **b** time spent in each unit (in minutes; plotted in log scale), **c** retention interval between final study session and unit quiz (in minutes; plotted in log scale), **d** unit quiz grade, **e** number of activities completed in each unit, and **f** mean time between sessions during study (in minutes; plotted in log scale). Dotted line represents the mean of the distribution.

From the data available we extracted multiple measures: pretest scores (a measure of previous knowledge), learning outcomes (quiz grades and exam grades), and study behaviors (spacing, study time, retention interval, and activity completion). Visual inspection of these measures, as indicated in Fig. 1, shows good distribution and variability.

### Prior knowledge

All students were given a pretest on questions related to general psychology prior to beginning the course. The test consisted of 20 true/false questions. The average score was 11% (*M* = 10.93, SD = 3.46, range 2–20). Students with higher prior knowledge generally did better in the course: their average unit quiz scores were higher, *r* = 0.19, *p* < 0.001, as were their final exam scores, *r* = 0.20, *p* < 0.001. Although these correlations are small, we included pretest score as a predictor variable in all the regression analyses reported below.

### Learning outcomes

Participants’ learning outcomes were measured by performance on 11 unit quizzes and the final exam (*M* = 27.72, SD = 5.50, range 4–35). In all analyses for which these learning outcomes were the criterion variable, pretest score was included as a predictor. Individuals’ average unit quiz score and final exam score were positively correlated, *r* = 0.69, *p* < 0.001.

### Study behaviors

Each time a page was loaded or a response to an activity was made, the data (e.g., page information, response) were captured together with a timestamp. From these logs, we created our variables to represent spacing, time spent, retention interval, and activity completion rate.

We defined spacing as the number of sessions that students took to complete a given unit. The more sessions they had, the more spaced we considered learning to be. On average, students took 4.08 sessions to complete each unit (SD = 3.17, median = 3).

From the timestamps, we could also calculate the total amount of study time. We calculated this by summing the duration of all student sessions. The duration of each session was calculated by calculating the time difference between each event in that session. Students took an average of 2 days to complete each unit (SD = 3 days, median = 1 day).

By calculating the difference between the timestamp on the last studied page or last activity worked on and when a student began the unit exam, we could calculate the retention interval (*M* = 45.73 h; SD = 146.49 h; median = 26.12 min).

Finally, activity completion rate was defined as the number of activities completed in a given unit. Activities included fill-in-blank, multiple-choice, and drag-and-drop questions that were interpolated throughout each unit.

### Inferential statistics

In all our regression models, we included pretest grade and time spent in unit as predictors, and included unit and student as random factors. We included students’ pretest score to control for differences in prior knowledge. We included time spent in unit to control for differences in total study time, so that the number of sessions was not just about spending more study time. Including unit as a random factor controls for differences between units (e.g., some units may be longer or contain more content than others); including student as a random factor not only controls for differences between students in their tendencies to space, but also to interpret the effect as a within-participant effect. All tests of significance are two-tailed. All predictors were normalized by *z*-scoring the raw values. See Supplementary Materials for the full summary statistics of the regression analyses.

### What is the relationship between spacing and quiz performance?

Our main question was whether self-regulated spacing was positively related to quiz performance. We defined learning as unit quiz performance and spacing as the number of sessions a student spent studying that unit. We focus on unit quiz performance rather than final exam performance because students are unlikely to approach each of the 11 units with the same spacing; rather, some units might be studied in fewer sessions, and others might be studied in more sessions. One strength of our approach is that we can therefore examine the relationship between spacing and learning within participants.

In our regression model, we predicted quiz performance from spacing, and to control for possible moderating effects of different delays between study and test, we also included retention interval. We also controlled for pretest grade and time spent in unit, and included student and unit as random factors. Additionally, because prior studies have shown that the optimal spacing interval depends on the retention interval^13^, we also included an interaction term (number of sessions and retention interval). In other words, how much a person spaces their study can vary from unit to unit, and we are interested in examining whether relatively greater spacing is related to better learning.

Results (see Supplementary Table 1) revealed that spacing was a significant predictor of unit quiz grades, *β* = 0.10, SE = 0.02, *t*(6593.84) = 6.41, *p* < 0.001. Over and above the fact that some people space more than others, greater spacing of practice is related to better performance on the unit quiz. Moreover, although there was also a significant main effect of retention interval with shorter retention intervals being associated with higher quiz grades, *β* = −0.07, SE = 0.01, *t*(6664.91) = −5.19, *p* < 0.001, there was not a significant spacing by retention interval effect, *β* = 0.02, SE = 0.01, *t*(6512) = 1.28, *p* > 0.20. Spacing was better for quiz performance, even at short retention intervals.

A similar regression model was conducted to predict final exam grade and showed the same pattern of results: students who spaced their study more also performed better in the final exam, *β* = 0.13, SE = 0.05, *t*(727) = 2.76, *p* < 0.01, controlling for total time spent in the course, retention interval, and pretest score.

### Are learners who score higher on the final exam more likely to have spaced their study?

The prior analysis found that when students used more spacing in their learning, this behavior was related to better quiz performance. Now, we ask a between-participant question: how does this behavior vary between students? Specifically, are the better students the ones who are more likely to space their learning? In the absence of any information about prior GPA or other standardized tests, we used students’ final exam grades as a proxy for ability level. We acknowledge that the final exam grade is potentially confounded with actual spacing use during the course. To account for this possibility we also used pretest score to examine differences in student ability and found similar results. One issue with the pretest measure is that it was a substantially shorter assessment that focused on a reduced number of topics, and there was lower variability in pretest score compared to exam grade, which reduces our statistical power.

**Fig. 2:**
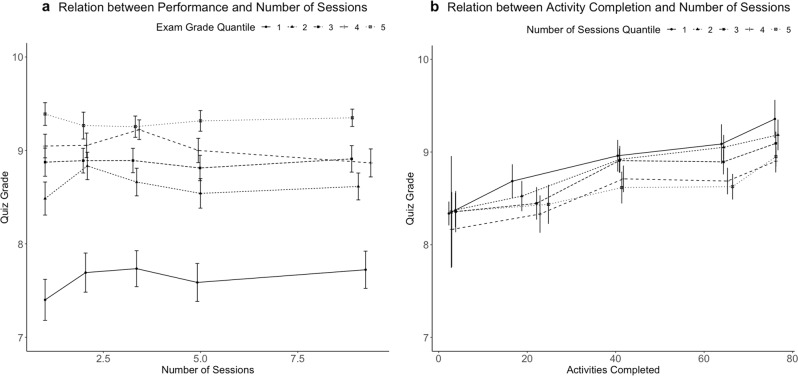
Plots of raw binned data. Plots of raw, binned data showing **a** relationship between spacing (defined as number of study sessions completed) and unit quiz grade as a function of final exam grade and **b** relationship between activities completed and unit quiz grade as a function of spacing. The error bars represent the 95% confidence interval. Note that in this figure we plot raw values for all variables. However, our analyses include covariates that are not taken into account in these graphs.

The left panel in Fig. 2 depicts mean quiz performance as a function of number of sessions completed and exam grade quantile. The plot suggests that the benefit of completing more sessions, i.e., spacing study more, is related to better quiz grades, particularly for students with lower exam grade.

To further investigate this pattern, we conducted a regression to predict the number of sessions spent in a unit from students’ final exam grade, controlling for pretest grade and time spent in unit. The results (see Supplementary Table 2) reveal that those students who performed better on the final exam were also more likely to space their study more, *β* = 0.06, SE = 0.02, *t*(787.7) = 2.82, *p* = 0.005.

**Fig. 3:**
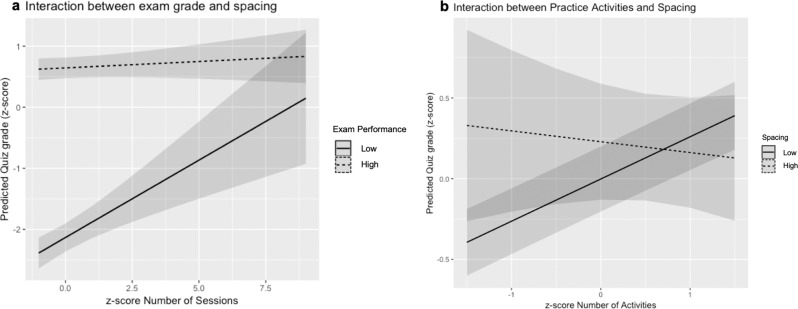
Plots of regression marginal effects for the interactions. Plots of marginal effects of the interactions in the regression models showing **a** the relationship between spacing (defined as number of study sessions) and unit quiz performance, by participant ability (defined by final exam score; the dotted line represents 1 SD above the mean final exam score; the solid line represents 1 SD below the mean final exam score) and **b** the relationship between number of activities completed and unit quiz grade, by spacing (the dotted line represents 1 SD above mean spacing; the solid line represents 1 SD below mean spacing). The shading represents 95% confidence intervals. These plots use model predictions to depict the effect that the interaction between two predictors has on the outcome variable controlling for the other predictions included in the model (see text for details).

Given that students of different ability levels choose to use spacing to differing degrees, we conducted another regression analysis to examine whether the relationship between spacing and quiz performance interacted with ability level, predicting quiz grade from spacing, final exam grade, and their interaction. Again, we also controlled for pretest grade and time spent in unit, and included student and unit as random factors. The results (see Supplementary Table 3) reveal that while there was still an overall benefit of spacing, *β* = 0.08, SE = 0.01, *t*(5644) = 5.24, *p* < 0.001, there was also a significant spacing by final exam grade interaction, *β* = −0.04, SE = 0.01, *t*(6017) = −3.12, *p* = 0.002. The left panel of Fig. 3 depicts the marginal effects of the interaction in the model by using both the final exam grade and spacing to predict quiz performance, controlling for pretest scores and total time spent studying. Spacing was related to higher quiz performance for students with lower ability but not for students with higher ability levels.

### Do learners with different practice levels make different spacing decisions, and do these matter for learning?

Prior studies have demonstrated benefits of active learning on outcomes^25,26,44^, but does the distribution of study relate to how students use their study time? In other words, when students space, are they also likely to engage in more active practice? We conducted a regression analysis (see Supplementary Table 4) predicting spacing of a unit from the number of activities completed in that unit. We controlled for pretest grade and time spent in unit, and included student and unit as random factors. The results (see Supplementary Table 4) revealed that students who completed more activities also tended to space their learning more, *β* = 0.35, SE = 0.01, *t*(4828) = 28.06, *p* < 0.001.

Second, does engaging in active learning attenuate or amplify the benefits of spacing? The right panel in Fig. 2 depicts mean quiz performance as a function of activity completion and number of sessions completed (binned as quantiles). This visualization suggests that the positive relation between spacing and quiz scores is particularly high for those who are now completing many activities. To further investigate this pattern, we conducted a regression analysis predicting quiz performance from spacing, number activities completed, and their interaction. Again, we also controlled for pretest grade and time spent in unit, and included student and unit as random factors. In this analysis (see Supplementary Table 5), we found a significant spacing by activities interaction, *β* = −0.03, SE = 0.01, *t*(6415) = −2.71, *p* = 0.007. This interaction is illustrated in the right panel of Fig. 3, which plots the marginal effects of the interaction by using both the number of activities and spacing to predict quiz performance, controlling for pretest scores and total time spent studying. When students completed fewer activities, the positive relationship between spacing and quiz performance was greater than when they completed more activities.

## Discussion

Research shows that spacing enhances learning in the laboratory settings in which spacing is imposed by an experimenter, but does it work in educational settings where spacing is chosen by the learner? Learning is largely self-regulated. This is especially true in post-secondary education, and it is increasingly true more generally, as the information becomes increasingly available at our fingertips^45^. For example, in college, students are expected to manage their own study time outside of lecture time; in high schools, there may be an online component in a course; in the workplace, learning may take place in the absence of any instructor or formal educational settings at all.

Thus, if learning is largely self-regulated, then to better understand how spacing relates to learning it is important to examine how people naturally use spacing in self-regulated situations, i.e., “in the wild”. This is particularly the case when the goal is to prescribe how learners should incorporate spacing into their own studies^46^. In the present study, we examined learners’ self-regulated spacing behaviors in an online course and how their choices were related to their learning outcomes. This research combines and contributes to our understanding of what type of learning decisions students naturally make during the span of a semester-long course, the individual differences that relate to these decisions, and how decisions are related to learning outcomes.

Overall, we found that spacing is related to better learning outcomes. Perhaps better students are more likely to space their learning. This could be, for example, due to better organization or knowledge of spacing benefits. Indeed, we found that higher ability students were more likely to space their study than lower ability students. However, if this were the whole story, the results might not be very interesting; other studies have demonstrated that lower ability students are more likely to engage in less effective learning strategies than high ability students^34^.

Importantly, although we cannot completely eliminate the existence of a third variable that explains these findings (e.g., motivation, life events), our analytic approach does allow us to more closely relate students’ decisions to their learning outcomes. We achieve this by comparing, for the same student, how different decisions across the different units related to their learning outcomes. That is, rather than giving students a single set of materials and assessing how they choose to space or mass the study of that set, we examined data from a course in which students studied and took quizzes on 11 different units. We therefore probed how spacing decisions and quiz performance varied within each individual. These analyses reveal a more complex and interesting story. We found that greater spaced study of a unit is associated with higher scores on that unit quiz. Moreover, this relationship between spacing and quiz performance is particularly pronounced for lower-ability students (those who had lower final exam scores), controlling for prior knowledge (pretest score).

We observed a similar pattern of findings when analyzing how students use practice testing during the course. Consistent with previous research^25,26,44^, we found that, overall, completing more practice activities in a unit was associated with better quiz grades for that unit. Importantly, although students are more likely to space their study when they complete more activities, the relationship between spacing and quiz grades is more pronounced when students completed less activities. In other words, spacing out study appears to buffer students against the negative effects of otherwise-passive learning.

One possible explanation for these findings is that completing activities and spacing may, at least partially, enhance learning via the same mechanism: retrieval. Completing the activities involves retrieval of prior knowledge and the generation of connections between new knowledge and prior knowledge^47,48^. Spacing promotes forgetting, which then fosters retrieval when one returns to a unit after a break^49–51^. If one strategy already engages a particular pathway, then we would not expect a purely additive effect of introducing another strategy that also engages the same pathway. However, it means that there are two methods that can get students to the same learning outcome: for those students who do not engage in as many activities during learning, they can instead space out their learning more to obtain the same benefits. Those who do neither, however, are likely to underperform. As previous studies have shown, lower ability students are often less likely to spontaneously engage in active learning processes^39–42^, which might be related to why lower ability students are more likely to benefit from spacing, as shown in the present study.

In sum, by analyzing how natural learning decisions of spacing and practice relate to students’ learning outcomes in an online course, we were able to demonstrate that spaced study is related to better learning outcomes, even in situations where students decide to do it. Moreover, we found that spacing is related to learning benefits, particularly for those students who are not taking advantage of other opportunities (e.g., activities) to improve learning.

The spacing effect is one of the most robust effects in the cognitive psychology literature. It has been demonstrated to be effective when learners have the schedule of learning imposed upon them in laboratory settings; in the present study, we show that it is related to positive learning outcomes in real semester-long courses, even when spacing is self-regulated. Moreover, the present study reveals nuances in who chooses it, who benefits from it, and how spacing study might in fact be an alternative method of engaging active learning processes.

## Methods

### Ethics

Data collection for the MOOC was approved under Carnegie Mellon University Institutional Review board (CMU IRB) protocol #HS11-351. The DataShop repository and its use is approved under CMU IRB protocol #IRBSTUDY2015_00000236. As the data are archival and anonymous, there was no written informed consent required. As per Datashop requirements, all available data was verified for appropriate student participation agreement and IRB oversight, and students provided consent to have their data analyzed.

### Course and participants

Data was drawn from a psychology MOOC; the data were retrieved from DataShop, an open learning repository for educational data^52^. The data we analyzed are freely available through DataShop (https://pslcdatashop.web.cmu.edu/), dataset 863. All code used for analyses is available in github (https://github.com/pcarvalh/Self-regulated-spacing-online-class).

The course was offered through Coursera by an instructor from Georgia Institute of Technology in 2013 (Prof. Anderson Smith). The course was fully open to the general public. The course was 12 weeks long, beginning March 25th, 2013 and ending June 15th, 2013. Students could sign up through Coursera. A total of 5615 students enrolled and agreed to have their data included for research purposes. Of these, 747 completed the course (i.e., completed the final exam), and we constrained our analyses to the data from these students.

Each week, students were expected to watch video lectures, and complete the related online textbook unit. At 8:00 am EDT on the Friday of each week, a multiple-choice quiz was released, testing students on the concepts from that week. This occurred every week, with the exception of the final week, where there was a final exam instead of a weekly quiz. These quizzes were due by 8:00 am EDT the following Friday. All students included in the analyses completed at least one of the quizzes, and most students included in the analyses completed all 11 quizzes (*N* = 639).

### Course materials

The course was comprised of 12 units, plus an initial “learning strategies” unit. Each unit consisted of online textbook (including additional short videos and activities) and video lectures (created by the instructor and distributed through Coursera). We did not have access to the videos and for the purpose of our research questions, we focused on students’ engagement with the online textbook only. The entire course consisted of a total of 214 pages, 645 activities, 187 images, and 43 video lectures.

### Assessments

Students completed a quiz for each unit except unit 12 (which was covered only on the final exam). The quizzes are worth 30% of the students’ final grade and the lowest quiz score was automatically dropped. Quizzes were completed in Coursera. Quizzes had a completion timeline (each week a quiz was made available on Friday and closed the following Friday), were not timed, but each student could only take each quiz once. Multiple students were granted extensions to complete the quizzes.

The final exam (worth 40% of the final grade) covered material from the entire course. It was released on June 10th, 2013 and stayed open until June 15th, 2013. The remaining 30% of the final grade was based on written assignments. Students had to score at least 70% overall to pass the course.

## Supplementary information

Supplemental Materials

Reporting Sum

## Data Availability

The dataset analyzed during this current study is freely available through DataShop (https://pslcdatashop.web.cmu.edu/), dataset 863.

## Code availability

All code used for analyses is available in github (https://github.com/pcarvalh/Self-regulated-spacing-online-class).
