# Supplementary material for: Self-regulated spacing in a massive open online course is related to better learning
Source: NPJ Sci Learn. 2020 Mar 16;5:2. doi: 10.1038/s41539-020-0061-1 (PMC7076029; doi:10.1038/s41539-020-0061-1)
Supplement: Supplementary file 1 — Supplemental Materials [file 41539_2020_61_MOESM1_ESM.pdf]

Supplementary Materials

Supplementary Table 1

Regression Summary Statistics, Predicting Unit Quiz Grades from Spacing and Retention Interval

**Predictor**	**β**	**SE**	***t***	**df**	***p***
Spacing (number of sessions)	0.10	0.02	6.41	6593.84	< .001
Retention interval	-0.07	0.01	-5.19	6664.91	<.001
Time spent	-0.03	0.01	-2.07	6284.03	.039
Pretest grade	0.13	0.02	5.46	671.27	<.001
Spacing x Retention interval	0.02	0.01	1.28	6512.03	.203

Supplementary Table 2

Regression Summary Statistics, Predicting Spacing from Student Ability

**Predictor**	**β**	**SE**	***t***	**df**	***p***
Exam grade	0.06	0.02	2.82	787.7	.005
Time spent	0.32	0.01	37.36	6365	<.001
Pretest grade	-0.07	0.02	-3.12	739.1	.002

Supplementary Table 3

Regression Summary Statistics, Predicting Unit Quiz Grades from Spacing and Student Ability

**Predictor**	**β**	**SE**	***t***	**df**	***p***
Spacing (number of sessions)	0.08	0.01	5.24	5644	< .001
Exam grade	0.44	0.02	25.18	721.22	< .001
Time spent	-0.01	0.01	-1.15	6528	.250
Pretest grade	0.04	0.02	2.17	663.37	.030
Spacing x Exam grade	-0.04	0.01	-3.12	6017	.002

Supplementary Table 4

Regression Summary Statistics, Predicting Spacing from Activity Completion Rate

**Predictor**	**β**	**SE**	***t***	**df**	***p***
Number of activities	0.35	0.01	28.06	4828	< .001
Time spent	0.29	0.01	34.30	6333	<.001
Pretest grade	-0.05	0.02	-2.54	769.3	.011

Supplementary Table 5

Regression Summary Statistics, Predicting Unit Quiz Grades from Spacing and Activity Completion Rate

**Predictor**	**β**	**SE**	***t***	**df**	***p***
Spacing (number of sessions)	0.03	0.02	1.81	6559	.070
Number of activities	0.26	0.02	14.94	6079	<.001
Time spent	-0.03	0.01	-2.28	6326	.023
Pretest grade	0.13	0.02	5.79	666.2	<.001
Spacing x Activities	-0.03	0.01	-2.71	6415	.007
